# Integration of bioinformatics analysis, molecular docking and animal experiments to study the therapeutic mechanisms of berberine against allergic rhinitis

**DOI:** 10.1038/s41598-024-60871-4

**Published:** 2024-05-25

**Authors:** Gaoqing Luo, Ming Gao, Qinghua Lin

**Affiliations:** 1 Department of Otolaryngology, Fujian Provincial Governmental Hospital, Fuzhou, 350003 China; 2 Department of Otolaryngology, People’s Hospital of Changji Hui Autonomous Prefecture, Changji, 831100 China

**Keywords:** Berberine, Allergic rhinitis, Network pharmacology, Hub genes, Molecular docking, Computational biology and bioinformatics, Pharmacology, Target identification

## Abstract

Allergic rhinitis is a prevalent inflammatory condition that impacts individuals of all age groups. Despite reports indicating the potential of berberine in alleviating allergic rhinitis symptoms, the specific molecular mechanisms and therapeutic targets of berberine remain unclear. This research aims to explore the pharmacological mechanism of berberine in the treatment of allergic rhinitis through bioinformatic analyses and experimental validation. The research utilized public databases to identify potential targets of berberine. Furthermore, differentially expressed genes (DEGs) related to allergic rhinitis were pinpointed from the GSE52804 dataset. Through bioinformatics techniques, the primary targets were discovered and key KEGG and GO-BP pathways were established. To confirm the therapeutic mechanisms of berberine on allergic rhinitis, an OVA-induced allergic rhinitis model was developed using guinea pigs. We identified 32 key genes responsible for the effectiveness of berberine in treating allergic rhinitis. In addition, five central genes (Alb, Il6, Tlr4, Ptas2, and Il1b) were pinpointed. Further examination using KEGG and GO-BP pathways revealed that the main targets were primarily involved in pathways such as NF-kappa B, IL-17, TNF, and inflammatory response. Molecular docking analysis demonstrated that berberine exhibited strong affinity towards these five key targets. Furthermore, the expression levels of IL-6, TLR4, PTGS2, and IL-1β were significantly upregulated in the model group but downregulated following berberine treatment. This research has revealed the mechanism through which berberine combats allergic rhinitis and has identified its potential to regulate pathways linked to inflammation. These discoveries provide valuable insights for the development of novel medications for the treatment of allergic rhinitis.

## Introduction

Allergic rhinitis is a prevalent chronic respiratory condition worldwide. Common symptoms of allergic rhinitis include nasal congestion, sneezing, and nasal itching, significantly impacting individuals' quality of life^1^. While not life-threatening, this illness can lead to obstructive sleep apnea^2^. Additionally, allergic rhinitis is associated with numerous other conditions, such as asthma and allergic conjunctivitis^3^, and can exacerbate asthma^4^. Allergic rhinitis is initiated by specific IgE-mediated inflammation, with the involvement of various inflammatory cells such as mast cells, eosinophils, regulatory molecules, and cytokines in the inflammatory process^5^. Moreover, the levels of allergic mediators such as TNF-α, IL-6, and histamine were elevated in immunoglobulin E-stimulated inflammatory cells, further worsening the progression of allergic rhinitis^6,7^. Recent research has shown that genetic regulations and various pathogenic factors play a role in the onset and progression of allergic rhinitis^1^. However, the etiology of allergic rhinitis remains incompletely elucidated. While there are medications like nasal anticholinergic drugs, ketones, glucocorticoids, and antihistamines available for its treatment, many patients experience suboptimal outcomes or treatment failures^8^. Hence, it is imperative to develop new and efficacious therapies for allergic rhinitis.

Berberine, derived from Rhizoma coptidis (Huanglian), is a key bioactive compound^9^. Recent research has shown that berberine possesses pharmacological properties that target a range of illnesses, such as neurological, cardiovascular, metabolic, gastrointestinal, and cancer-related disorders^10^. Berberine has been shown to inhibit the activation of the NLRP3 inflammasome through mitophagy^11^. Additionally, berberine has garnered attention for its potential in treating respiratory conditions. For instance, berberine demonstrates a beneficial impact on ovalbumin-induced asthma by suppressing the NF-кB signaling pathway^12^. Research has indicated that berberine can alleviate chronic obstructive pulmonary disease induced by cigarette smoke extract by modulating the TGF-β1/Smads signaling pathways^13^. Moreover, berberine mitigates bleomycin-induced pulmonary fibrosis by blocking NF-кB-mediated TGF-β activation^14^. A prior study indicated that berberine has the potential to reduce allergic inflammation in a mouse model of allergic rhinitis^15^. Nevertheless, the specific mechanism by which berberine acts against allergic rhinitis remains unclear.

**Figure 1:**
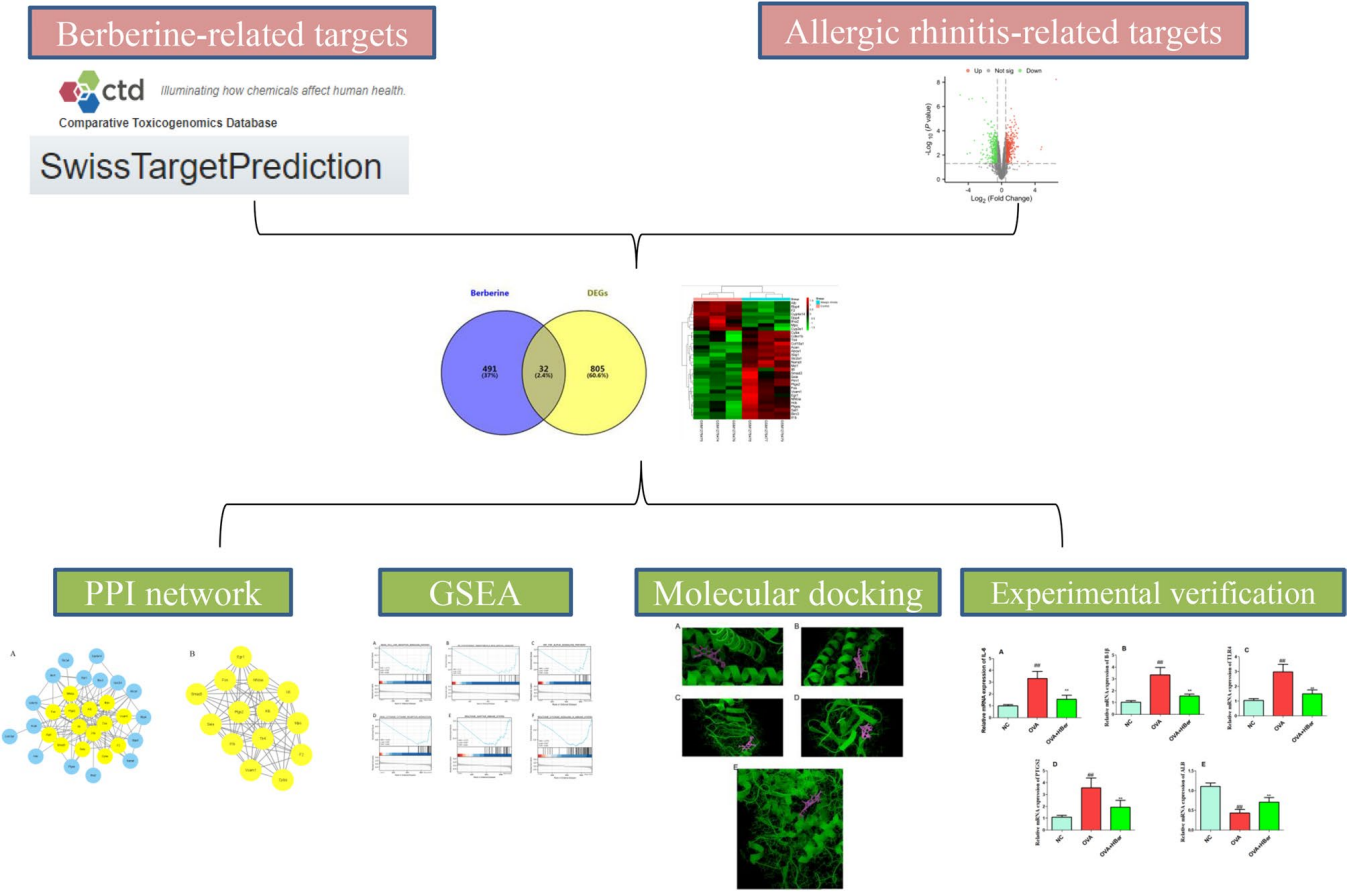
Workflow of the present study.

Network pharmacology represents an innovative strategy for identifying key drug targets and elucidating the underlying mechanisms associated with human diseases^16^. In the present study, network pharmacology was utilized to investigate the molecular mechanisms responsible for the therapeutic effects of berberine in the treatment of allergic rhinitis. The research methodology is visually presented in Fig. 1.

## Materials and methods

### Collecting potential targets of berberine and allergic rhinitis

SwissTargetPrediction is an online tool designed to forecast the primary protein targets of small compounds^17^. The Comparative Toxicogenomics Database (CTD) contains vast collections of selected gene-chemical, gene-disease, and chemical-disease associations^18^. Target collection of berberine was performed using the SwissTargetPrediction (http://www.swisstargetprediction.ch/, assessed on 16 May 2023), and CTD (https://ctdbase.org/, assessed on 16 May 2023). The Gene Expression Omnibus (GEO) serves as a global public repository where vast amounts of next-generation sequence functional genomic data and high-throughput microarray are deposited by researchers worldwide^19^. The GSE52804 dataset was downloaded from the GEO database (https://www.ncbi.nlm.nih.gov/geo/, assessed on 17 May 2023) and used to collect pathological genes of allergic rhinitis. The GSE52804 dataset comprised 3 control group samples (mice challenged with saline) and 3 ovalbumin group samples (mice challenged with ovalbumin). Differential gene expression analysis between control and allergic rhinitis was conducted using the "limma" package (v 3.22.7), with a significance threshold of p < 0.05 and ∣log fold change∣ ≥ 0.5. Intersection genes targeted by berberine in allergic rhinitis were identified using the Venn tool. The results were visualized using volcano plots and heat maps generated by the "ggplot2" R package (v 3.3.6).

### Construction of a protein–protein interaction (PPI) network and identification of hub genes

The interaction network of berberine in treating allergic rhinitis was constructed and visualized using STRING (https://string-db.org, accessed on 18 May 2023) and Cytoscape software (v 3.7.2). Subsequently, the key clusters within the network were identified through the MCODE plugin in Cytoscape. Finally, the top 10 genes were chosen based on nine different topological analysis algorithms in cytoHubba, including MCC, degree, closeness, stress, betweenness, radiality, eccentricity, bottleneck, and EPC^20^. The screening of hub genes was conducted using the R package "UpSet".

### KEGG and GOBP enrichment analysis

Enrichment analyses for KEGG and GOBP were conducted using the functional annotation tool Metascape (https://metascape.org/, accessed on May 18, 2023) to explore the potential biological processes of berberine in treating allergic rhinitis. The identified genes were inputted into Metascape and analyzed for enrichment specifically in "Homo sapiens" with a significance level of p < 0.05. The results of the enrichment analysis were visualized using the clusterProfiler package.

### Gene set enrichment analysis (GSEA)

In this study, the Gene Set Enrichment Analysis (GSEA) was performed to further explore the potential biological functions of predetermined genes in allergic rhinitis^21^. The Differentially Expressed Genes (DEGs) were identified between 3 control groups and 3 allergic rhinitis groups in the GSE52804 dataset. Significantly enriched genes were determined by False Discovery Rate (FDR) < 0.25 and p.adjust < 0.05.

### Molecular docking analysis

The current study utilized molecular docking to investigate the potential binding mode of active compounds with a large molecular receptor. Hub genes were chosen for the docking analysis, and the 3D structures of target proteins (Alb, Il6, Il1b, Tlr4, and Ptgs2) were sourced from the PDB database (https://www.rcsb.org, assessed on 20 May 2023) with a crystal resolution < 3 Å. Initially, PyMOL software was employed to eliminate solvents and organics from the protein structures. Subsequently, AutoDock software (v 1.5.6) was utilized for the molecular docking analysis, with the results visualized using PyMOL software (v 1.0.0).

### Establishment of ovalbumin (OVA)-induced allergic rhinitis model

40 guinea pigs (210–270 g and 6–7 weeks old) were acquired from the Guangdong Institute for Drug Control. The animals were provided with unlimited access to tap water and food, and were housed in a climate-controlled environment (with a relative humidity of 45–65% and a temperature range of 21–25 °C) under a 12-h light/dark cycle. The experimental procedures involving the animals were approved by the Ethics Committee of Fujian Provincial Government Hospital and were carried out in accordance with the ARRIVE guidelines.

Following a one-week acclimation period, the animals were divided into five groups, each consisting of 8 members: the normal control group (NC), the group with OVA-induced allergic rhinitis (OVA), the OVA group of guinea pigs treated with berberine at a dosage of 25 mg/kg (OVA + LBer), the OVA group of guinea pigs treated with berberine at a dosage of 50 mg/kg (OVA + MBer), and the OVA group of guinea pigs treated with berberine at a dosage of 100 mg/kg (OVA + HBer). The allergic rhinitis model was established by sensitizing and stimulating the guinea pigs with OVA, following a method described in a previous study^22^. In short, the guinea pigs in the OVA group were sensitized by receiving OVA (25 μg/guinea pig) injections into their peritoneal cavity three times per week for a period of two weeks. Following this initial sensitization phase, a solution containing OVA (100 μg/guinea pig) was dropped daily into the nasal passages of the guinea pigs for a week using micropipettes. The NC group received repeated sensitization with normal saline. Berberine (25, 50, or 100 mg/kg body weight) was orally given to guinea pigs with OVA-induced allergic rhinitis daily from day 21 to day 28. The dosage selection was based on a previous study^23^. The NC and OVA groups received only saline during the same procedure.

### Evaluation of rhinitis symptoms

Following the final treatment, the animals were placed in individual cages for a 30-min observation period, during which the number of times the guinea pigs rubbed and sneezed was documented.

### Quantification of levels of inflammatory cytokines and OVA-specific IgE in the serum

The guinea pigs were anesthetized with pentobarbital sodium (40 mg/kg) intraperitoneally 24 h after symptom evaluation. Blood was collected via intraorbital venipuncture, followed by serum separation through centrifugation at 1000*g* for 10 min. The levels of IL-6, IL-1β, IL-17, TNF-α, and OVA-specific IgE in the serum were quantified using enzyme-linked immunosorbent assay (ELISA) according to the manufacturer's instructions (ThermoFisher, MA, USA).

### Quantitative real-time polymerase chain reaction (qRT-PCR)

Nasal mucosa tissues were processed to extract total RNA using TRIzol Reagent (Invitrogen, CA, USA) following the manufacturer's instructions. The purified RNA (2 µg) was converted into cDNA with cDNA synthesis kits (Invitrogen, CA, USA), followed by qRT-PCR analysis on a StepOne Real-time PCR system (Applied Biosystems, CA, USA). mRNA expression levels were quantified using the 2^–ΔΔCt^ method with the primers listed in Table S1.

### Statistical analysis

R software (v 4.2.1) was utilized for the analysis and visualization of bioinformatics data. The limma package (v 3.52.2) was employed to identify DEGs, while enrichment analysis was conducted with the clusterProfiler package (v 4.4.4). The data from the experiment was analyzed using GraphPad Prism software (v 5.0.1). Results were presented as mean ± standard deviation (SD). Data between the two groups were compared using a t-test. A statistically significant difference was indicated by p < 0.05.

### Ethics approval and consent to participate

The animal experimental procedure received ethical approval from the Ethics Committee of Fujian Provincial Government Hospital and was conducted in adherence to the ARRIVE guidelines. All methods were performed in accordance with the relevant guidelines and regulations.

## Results

### Screening potential targets of berberine in the treatment of allergic rhinitis

**Figure 2:**
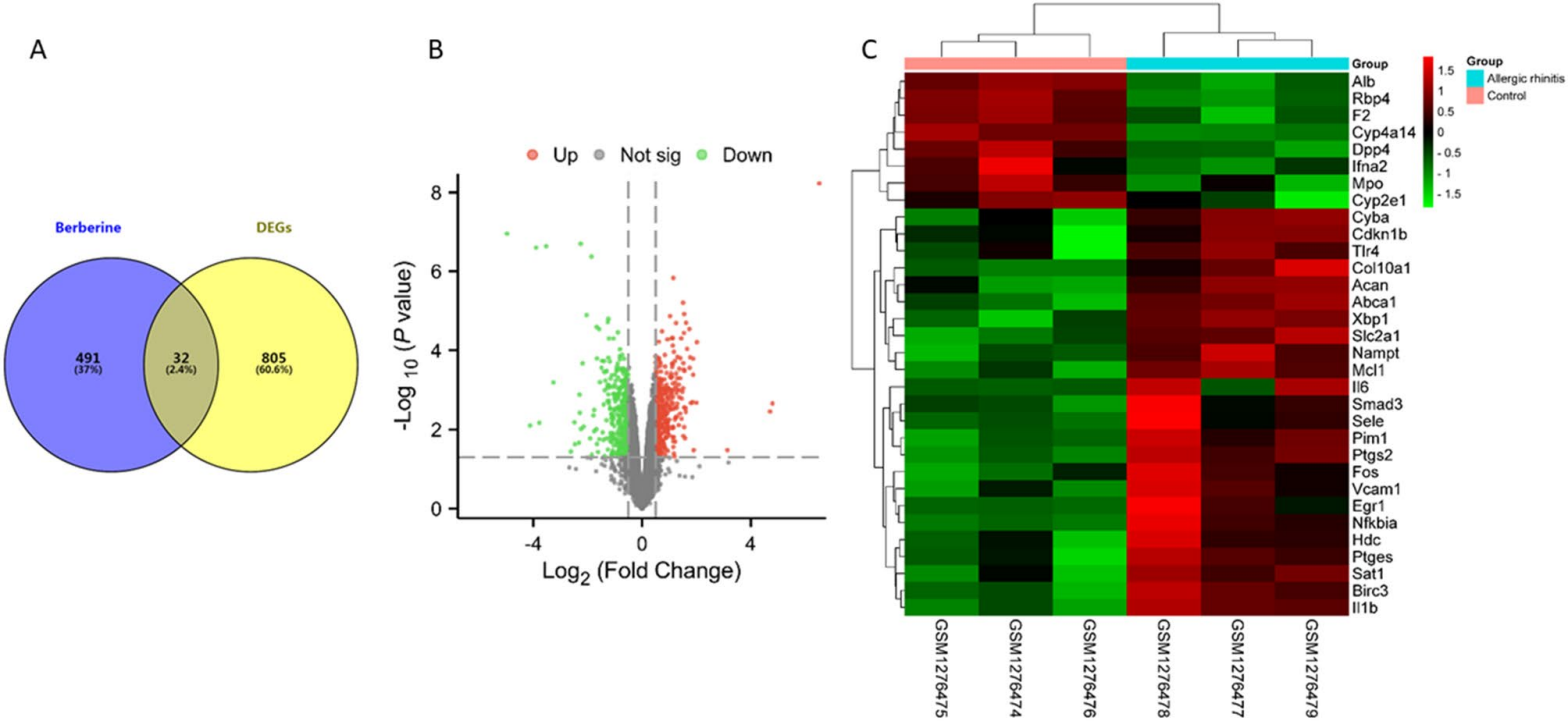
Collecting potential targets of berberine in the treatment of allergic rhinitis. (**A**) Venn diagram of DEGs and berberine-related genes. (**B**) The gene volcano map of DEGs. Green dots represented the down-regulated genes, red dots represented the up-regulated genes, and gray dots represented genes with no significant differences. (**C**) Clustered heat map of the 32 intersection genes.

Figure 2A illustrates the utilization of the Swiss Target Prediction and Comparative Toxicogenomics Database for the collection of 523 target genes associated with berberine. Additionally, 837 DEGs were extracted from the GSE52804 dataset. Subsequently, 32 genes were identified as potential targets of berberine in the treatment of allergic rhinitis through the intersection analysis (Fig. 2A and Table 1). The gene volcano plot and heat map depicting these 32 intersection genes are depicted in Fig. 2B,C.

**Table 1:** 32 intersection genes of berberine and DEGs in GSE52804.

Gategory	Gene symbol	log2 (fold change)	p value
Down-regulated	Il1b	− 2.19	p < 0.001
	Il6	− 1.37	p < 0.05
	Nampt	− 6.46E−01	p < 0.01
	Tlr4	− 5.10E−01	p < 0.05
	Cyba	− 6.60E−01	p < 0.01
	Smad3	− 5.21E−01	p < 0.05
	Acan	− 9.33E−01	p < 0.01
	Cdkn1b	− 5.28E−01	p < 0.05
	Col10a1	− 1.76	p < 0.01
	Fos	− 5.94E−01	p < 0.05
	Nfkbia	− 1.12	p < 0.01
	Ptgs2	− 1.3	p < 0.001
	Pim1	− 6.70E−01	p < 0.01
	Sele	− 1.04	p < 0.05
	Xbp1	− 5.39E−01	p < 0.01
	Ptges	− 5.42E−01	p < 0.01
	Slc2a1	− 5.95E−01	p < 0.01
	Mcl1	− 5.73E−01	p < 0.01
	Abca1	− 5.02E−01	p < 0.01
	Birc3	− 9.23E−01	p < 0.01
	Sat1	− 8.51E−01	p < 0.01
	Vcam1	− 6.98E−01	p < 0.05
	Egr1	− 5.61E−01	p < 0.05
	Hdc	− 6.24E−01	p < 0.05
Up-regulated	Ifna2	6.21E−01	p < 0.05
	Cyp2e1	6.40E−01	p < 0.05
	Cyp4a14	1.51	p < 0.001
	Alb	1.13	p < 0.001
	Mpo	1	p < 0.05
	Rbp4	1.74	p < 0.001
	Dpp4	5.19E−01	p < 0.01
	F2	5.27E−01	p < 0.01

### Analysis of the PPI network and identification of primary clusters

**Figure 3:**
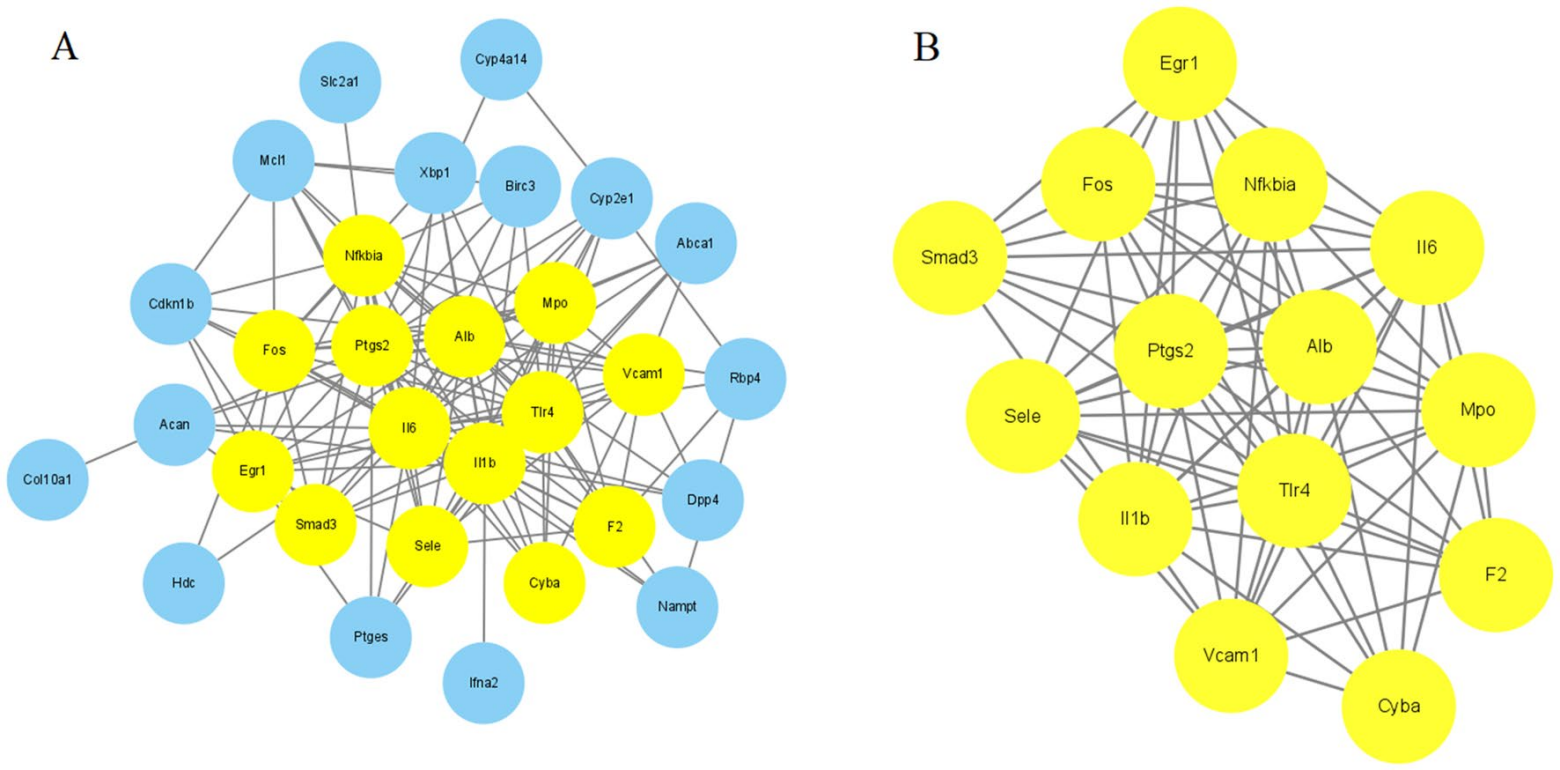
Analysis of PPI network and clusters. (**A**) PPI network of 32 intersection genes visualized by Cytoscape software. (**B**) Primary clusters are constructed based on the MCODE plugin.

Figure 3A illustrates the utilization of Cytoscape software in constructing a PPI network to visualize the interactions among the 32 overlapped genes. Furthermore, the MCODE plugin was employed to pinpoint 14 genes (Cyba, Nfkbia, Fos, Mpo, Il6, Il1b, Sele, Vcam1, F2, Tlr4, Alb, Egr1, Smad3, and Ptgs2) forming a primary cluster (Fig. 3B), potentially representing a crucial regulatory network for berberine in treating allergic rhinitis.

### KEGG and GO-BP enrichment analyses

**Figure 4:**
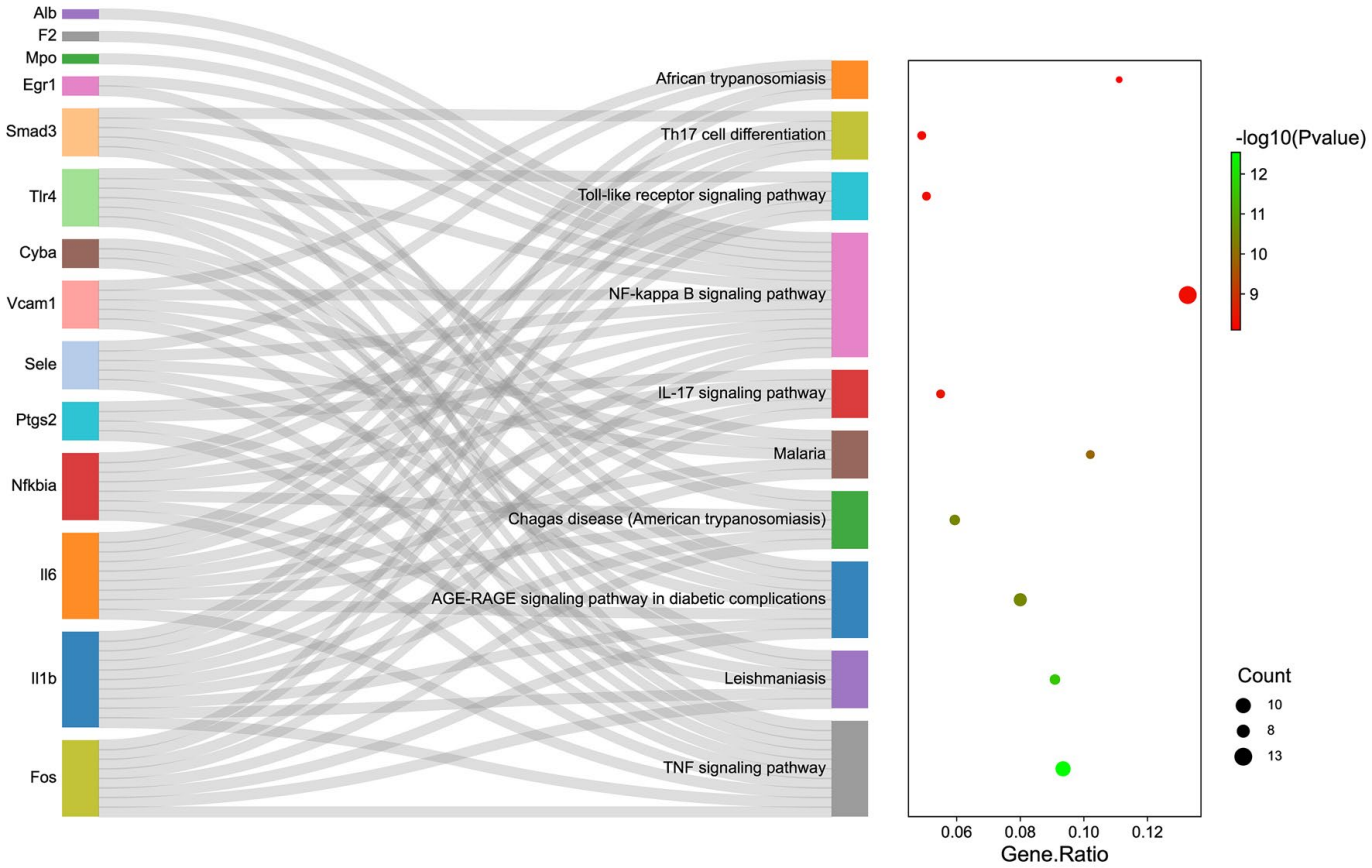
KEGG enrichment analysis. The Sankey map showed the relationship between targets and the top 10 KEGG pathways (left). The bubble diagram depicted the top 10 KEGG pathways (right).

**Figure 5:**
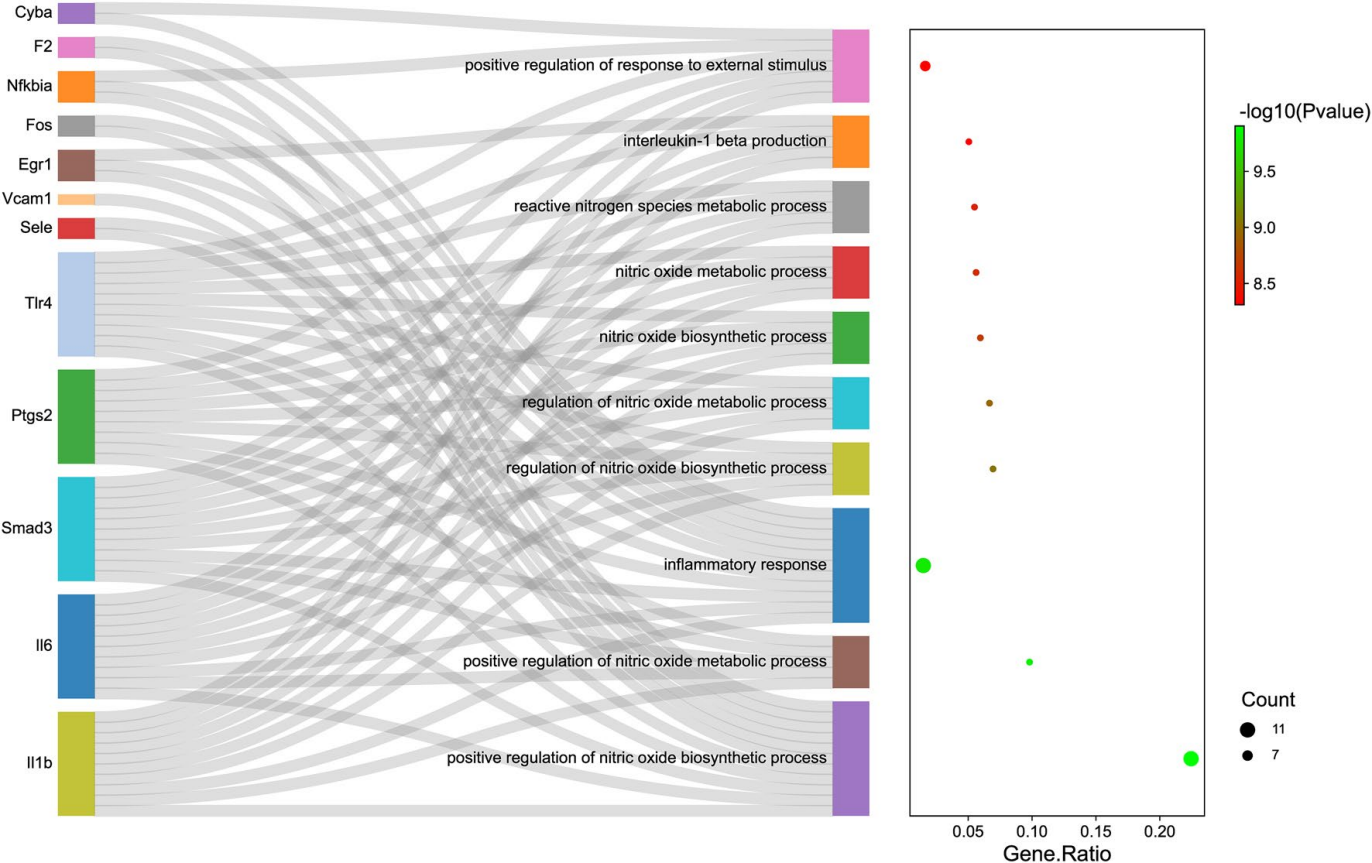
GO-BP enrichment analysis. The Sankey map showed the relationship between targets and the top 10 GO-BP terms (left). The bubble diagram depicted the top 10 GO-BP terms (right).

Enrichment analyses were conducted to further explore the biological functions of the potential genes of berberine in relation to allergic rhinitis. As shown in Fig. 4 and Table 2, KEGG enrichment indicated that the 14 core genes were mainly enriched in the TNF signaling pathway (Fig. S2), Leishmaniasis, AGE-RAGE signaling pathway in diabetic complications, Chagas disease, Malaria, IL-17 signaling pathway, NF-kappa B signaling pathway (Fig. S1), Toll-like receptor signaling pathway, Th17 cell differentiation, and African trypanosomiasis, etc. As presented in Fig. 5 and Table 3, GO-BP enrichment analysis revealed that the 14 core genes were significantly involved in positive regulation of nitric oxide biosynthetic process, positive regulation of nitric oxide metabolic process, inflammatory response, regulation of nitric oxide biosynthetic process, regulation of nitric oxide metabolic process, nitric oxide biosynthetic process, nitric oxide metabolic process, reactive nitrogen species metabolic process, interleukin-1 beta production, and positive regulation of response to external stimulus, etc.

**Table 2:** Top 10 KEGG pathways of berberine against allergic rhinitis.

Pathway	Gene ratio	p value	Genes	Count
TNF signaling pathway	0.093457944	p < 0.001	Fos/Il1b/Il6/Nfkbia/Ptgs2/Sele/Vcam1/Cyba/Tlr4/Smad3	10
Leishmaniasis	0.090909091	p < 0.001	Cyba/Fos/Il1b/Nfkbia/Ptgs2/Tlr4	6
AGE-RAGE signaling pathway in diabetic complications	0.08	p < 0.001	Egr1/Il1b/Il6/Smad3/Sele/Vcam1/Cyba/Fos	8
Chagas disease (American trypanosomiasis)	0.059405941	p < 0.001	Fos/Il1b/Il6/Smad3/Nfkbia/Tlr4	6
Malaria	0.102040816	p < 0.001	Il1b/Il6/Sele/Tlr4/Vcam1	5
IL-17 signaling pathway	0.054945055	p < 0.001	Fos/Il1b/Il6/Nfkbia/Ptgs2	5
NF-kappa B signaling pathway	0.132653061	p < 0.001	Il1b/Nfkbia/Ptgs2/Tlr4/Vcam1/Egr1/Smad3/Sele/Fos/Il6/Mpo/F2/Alb	13
Toll-like receptor signaling pathway	0.050505051	p < 0.001	Fos/Il1b/Il6/Nfkbia/Tlr4	5
Th17 cell differentiation	0.049019608	p < 0.001	Fos/Il1b/Il6/Smad3/Nfkbia	5
African trypanosomiasis	0.111111111	p < 0.001	Il1b/Il6/Sele/Vcam1	4

**Table 3:** Top 10 GO biological processes of berberine against allergic rhinitis.

Pathway	Gene ratio	p value	Genes	Count
Positive regulation of nitric Oxide biosynthetic process	0.224489796	p < 0.001	Il1b/Il6/Smad3/Ptgs2/Tlr4/Sele/Vcam1/Egr1/Fos/ Nfkbia/F2	11
Positive regulation of nitric Oxide metabolic process	0.098039216	p < 0.001	Il1b/Il6/Smad3/Ptgs2/Tlr4	5
Inflammatory response	0.01488498	p < 0.001	Cyba/F2/Il1b/Il6/Smad3/Nfkbia/Ptgs2/Sele/Tlr4/ Egr1/Fos	11
Regulation of nitric oxide Biosynthetic process	0.069444444	p < 0.001	Il1b/Il6/Smad3/Ptgs2/Tlr4	5
Regulation of nitric oxide Metabolic process	0.066666667	p < 0.001	Il1b/Il6/Smad3/Ptgs2/Tlr4	5
Nitric oxide biosynthetic process	0.05952381	p < 0.001	Il1b/Il6/Smad3/Ptgs2/Tlr4	5
Nitric oxide metabolic process	0.056179775	p < 0.001	Il1b/Il6/Smad3/Ptgs2/Tlr4	5
Reactive nitrogen species metabolic process	0.054945055	p < 0.001	Il1b/Il6/Smad3/Ptgs2/Tlr4	5
Interleukin-1 beta production	0.050505051	p < 0.001	Egr1/Il1b/Il6/Smad3/Tlr4	5
Positive regulation of response to external stimulus	0.016393443	p < 0.001	Cyba/Il1b/Il6/Smad3/Nfkbia/Ptgs2/Tlr4	7

### GSEA

**Figure 6:**
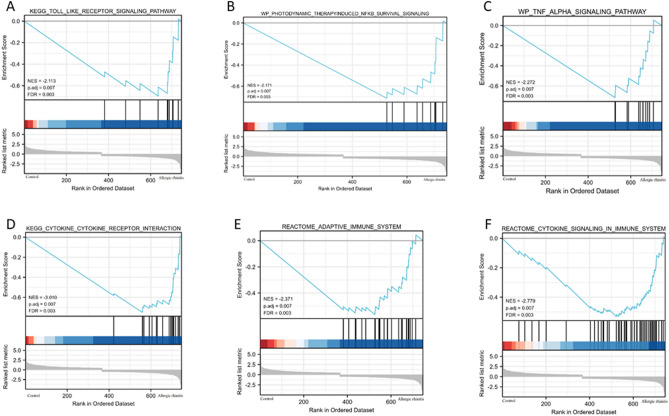
Enrichment analysis of DEGs based on GESA. (**A**) KEGG toll-like receptor signaling pathway, (**B**) WP photodynamic therapy induced NFKB survival signaling, (**C**) WP TNF-α signaling pathway, (**D**) KEGG cytokine-cytokine receptor interaction, (**E**) REACTOME adaptive immune system, and (**F**) REACTOME cytokine signaling in immune system.

Enrichment analysis using GSEA was conducted to further assess the associated pathways involved in the development of allergic rhinitis. As shown in Fig. 6, the findings revealed that the DEGs were significantly enriched in inflammation-related and immune-related pathways, including KEGG toll-like receptor signaling pathway, WP photodynamic therapy induced NFKB survival signaling, WP TNF-α signaling pathway, KEGG cytokine-cytokine receptor interaction, REACTOME adaptive immune system, and REACTOME cytokine signaling in immune system.

### Hub genes identification

**Figure 7:**
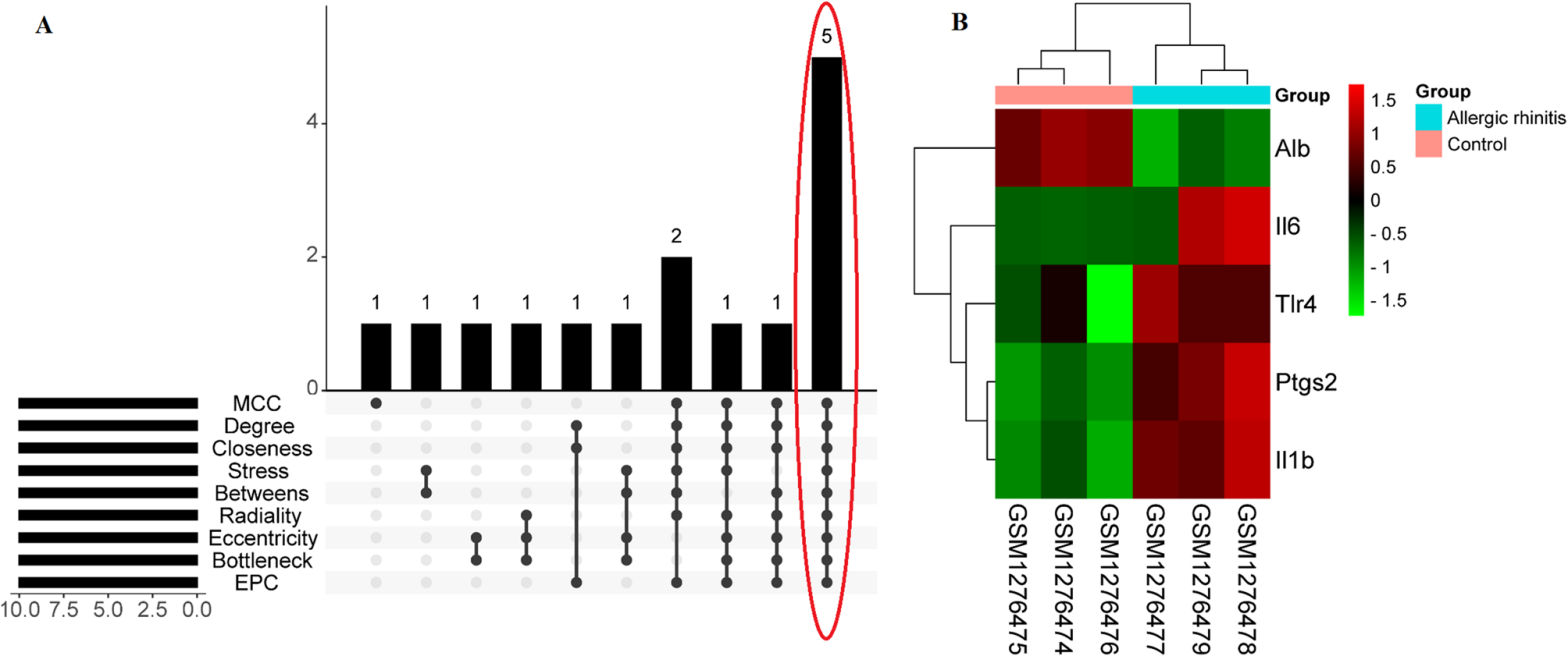
Hub genes identification. (**A**) Nine algorithms were carried out to identify hub genes based on the R package “UpSet”. (**B**) Heat map of five hub genes in the GSE52804 dataset.

The results from Table 4 show that nine topological analysis algorithms in cytoHubba selected the top 10 genes. Subsequently, the R package "UpSet" was utilized to pinpoint five hub genes—Alb, Il6, Il1b, Tlr4, and Ptgs2 (Fig. 7A). Additionally, a heatmap illustrating the mRNA expression levels of these five hub genes in the GSE52804 dataset was created for visualization purposes (Fig. 7B).

**Table 4:** Top 10 genes by 9 ranked methods in cytoHubba.

	Rank methods in cytoHubba								
	MCC	Degree	Closeness	Stress	Betweens	Radiality	Eccentricity	Bottleneck	EPC
Top 10 genes	Tlr4	Tlr4	Tlr4	Tlr4	Tlr4	Tlr4	Tlr4	Tlr4	Tlr4
	Alb	Egr1	Egr1	Alb	Alb	Alb	Alb	Alb	Egr1
	Nfkbia	Alb	Alb	Nfkbia	Nfkbia	Nfkbia	Acan	Acan	Alb
	Fos	Nfkbia	Nfkbia	Acan	Acan	Fos	Abca1	Abca1	Nfkbia
	Mpo	Fos	Fos	Fos	Fos	Smad3	Smad3	Smad3	Fos
	Ptgs2	Mpo	Mpo	Mpo	Cyp2e1	Mpo	Mpo	Mpo	Mpo
	Il6	Ptgs2	Ptgs2	Cyp2e1	Ptgs2	Ptgs2	Ptgs2	Ptgs2	Ptgs2
	Sele	Il6	Il6	Ptgs2	Il6	Il6	Il6	Il6	Il6
	Il1b	Il1b	Il1b	Il6	Il1b	Il1b	Il1b	Il1b	Il1b
	Vcam1	Vcam1	Vcam1	Il1b	Vcam1	Vcam1	Vcam1	Vcam1	Vcam1

### Molecular docking

**Figure 8:**
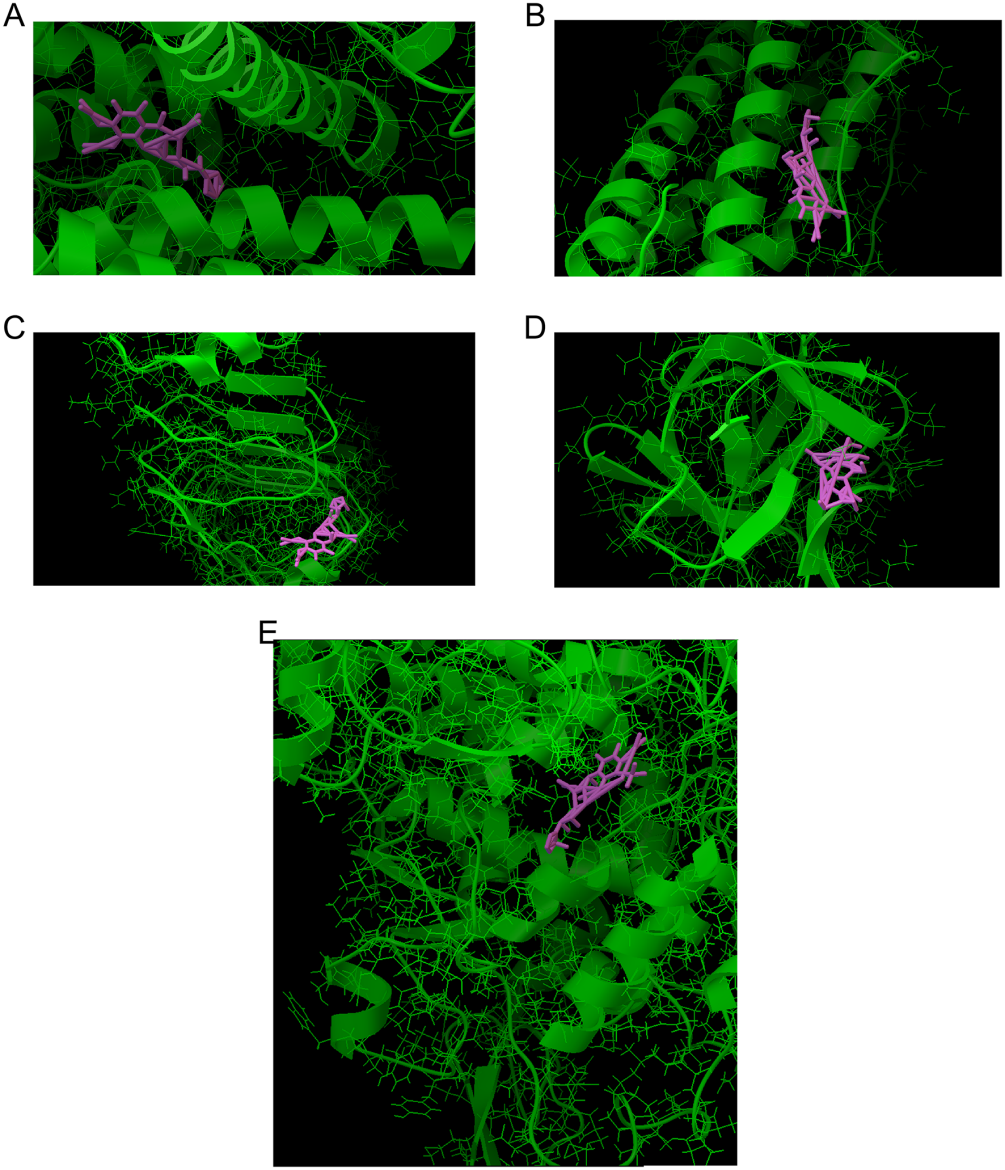
Molecular docking analysis of berberine binding to five hub targets. (**A**) berberine and Alb, (**B**) berberine and Il6, (**C**) berberine and Tlr4, (**D**) berberine and Il1b, (**E**) berberine and Ptgs2.

The study involved molecular docking analysis of berberine with five key genes (Alb, Il6, Il1b, Tlr4, and Ptgs2) to validate its potential targets against allergic rhinitis. The results from Table 5 and Fig. 8 revealed that berberine exhibited strong binding affinities with Il6 (− 9.93 kcal/mol), Tlr4 (− 8.0 kcal/mol), Il1b (− 8.5 kcal/mol), Ptgs2 (− 9.03 kcal/mol), and Alb (− 9.84 kcal/mol). These findings suggest that berberine effectively binds to Alb, Il6, Il1b, Tlr4, and Ptgs2 proteins, with binding energies below − 2 kcal/mol indicating their favorable interactions.

**Table 5:** Binding energies of berberine to the hub target proteins.

Target proteins	Binding energy (kcal/mol)				
	Alb	Il6	Tlr4	Il1b	Ptgs2
Berberine	− 9.84	− 9.93	− 8.0	− 8.5	− 9.03

### Berberine alleviates inflammatory response in OVA-induced allergic rhinitis

**Figure 9:**
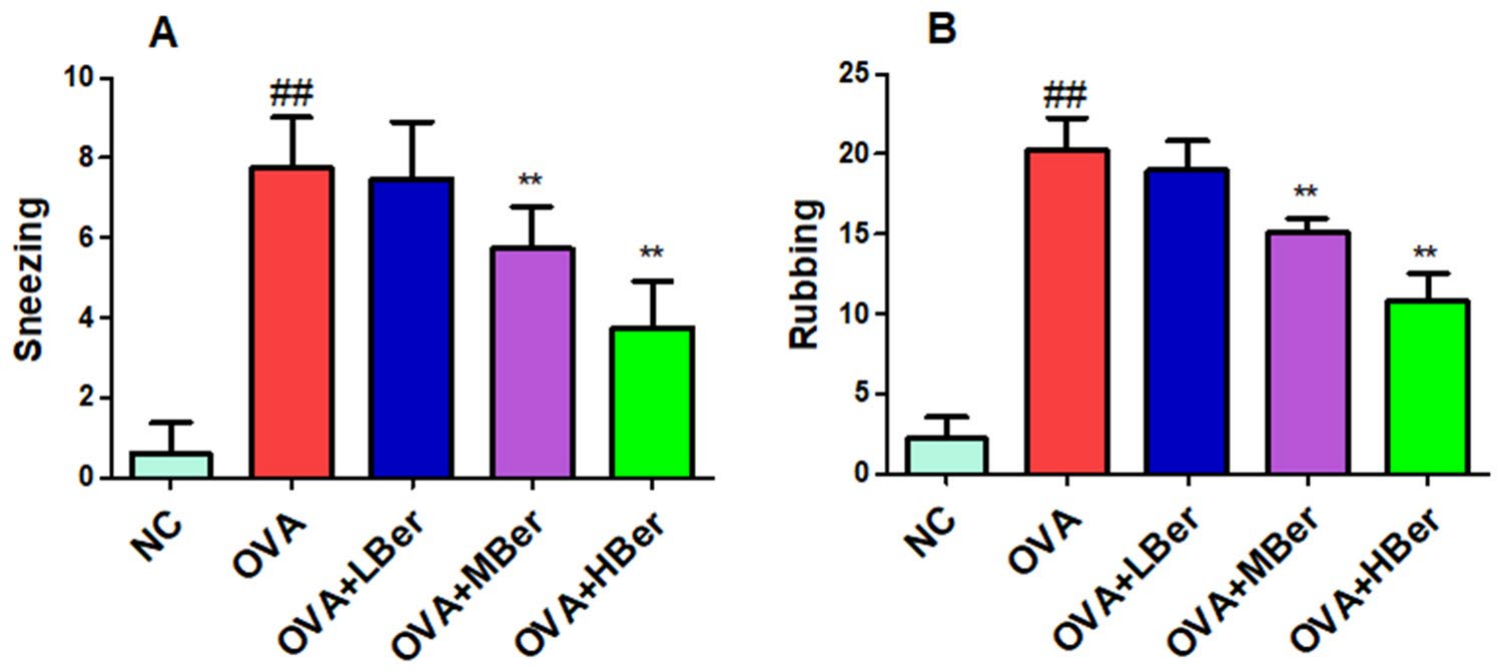
Berberine improved rhinitis symptoms in OVA-induced allergic rhinitis. The sneezing (**A**) and rubbing (**B**) times of animals were recorded. ^##^p < 0.01, the OVA group compared to the NC group; **p < 0.01, the therapeutic group compared to the OVA group. Allergic rhinitis was induced through an intraperitoneal injection of OVA (25 µg/guinea pig) three times a week for two weeks. The guinea pigs from the OVA group were treated with different doses of berberine (LBer: 25 mg/kg body weight; MBer: 50 mg/kg body weight; HBer: 100 mg/kg body weight).

**Figure 10:**
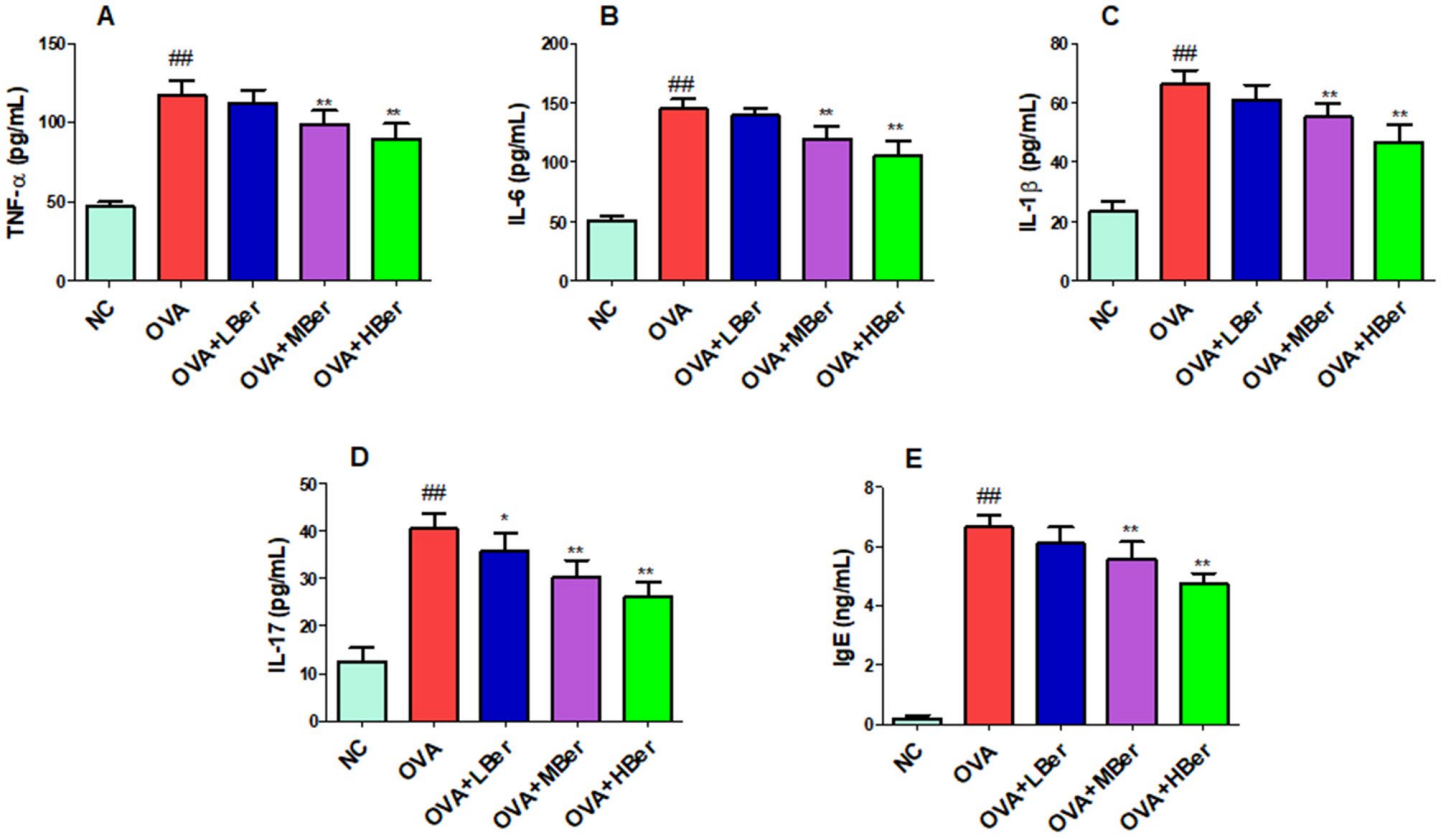
Berberine alleviates inflammatory response in OVA-induced allergic rhinitis. The TNF-α (**A**), IL-6 (**B**), IL-1β (**C**), IL-17 (**D**), and IgE (**E**) levels in serum. ^##^p < 0.01, the OVA group compared to the NC group; **p < 0.01, the therapeutic group compared to the OVA group. Allergic rhinitis was induced through an intraperitoneal injection of OVA (25 µg/guinea pig) three times a week for two weeks. The guinea pigs from the OVA group were treated with different doses of berberine (LBer: 25 mg/kg body weight; MBer: 50 mg/kg body weight; HBer: 100 mg/kg body weight).

Figure 9A,B demonstrate that treatment with berberine at doses of 50 and 100 mg/kg significantly reduced sneezing and rubbing in the OVA-induced allergic rhinitis group (p < 0.01). Furthermore, berberine (50 or 100 mg/kg) suppressed TNF-α, IL-6, IL-1β, IL-17, and IgE levels in the OVA-induced allergic rhinitis group (p < 0.01) (Fig. 10A–E). These results indicate that berberine can inhibit inflammatory responses in OVA-stimulated guinea pigs, aligning with the bioinformatics findings.

### Berberine inhibited hub genes expression in OVA-induced allergic rhinitis

**Figure 11:**
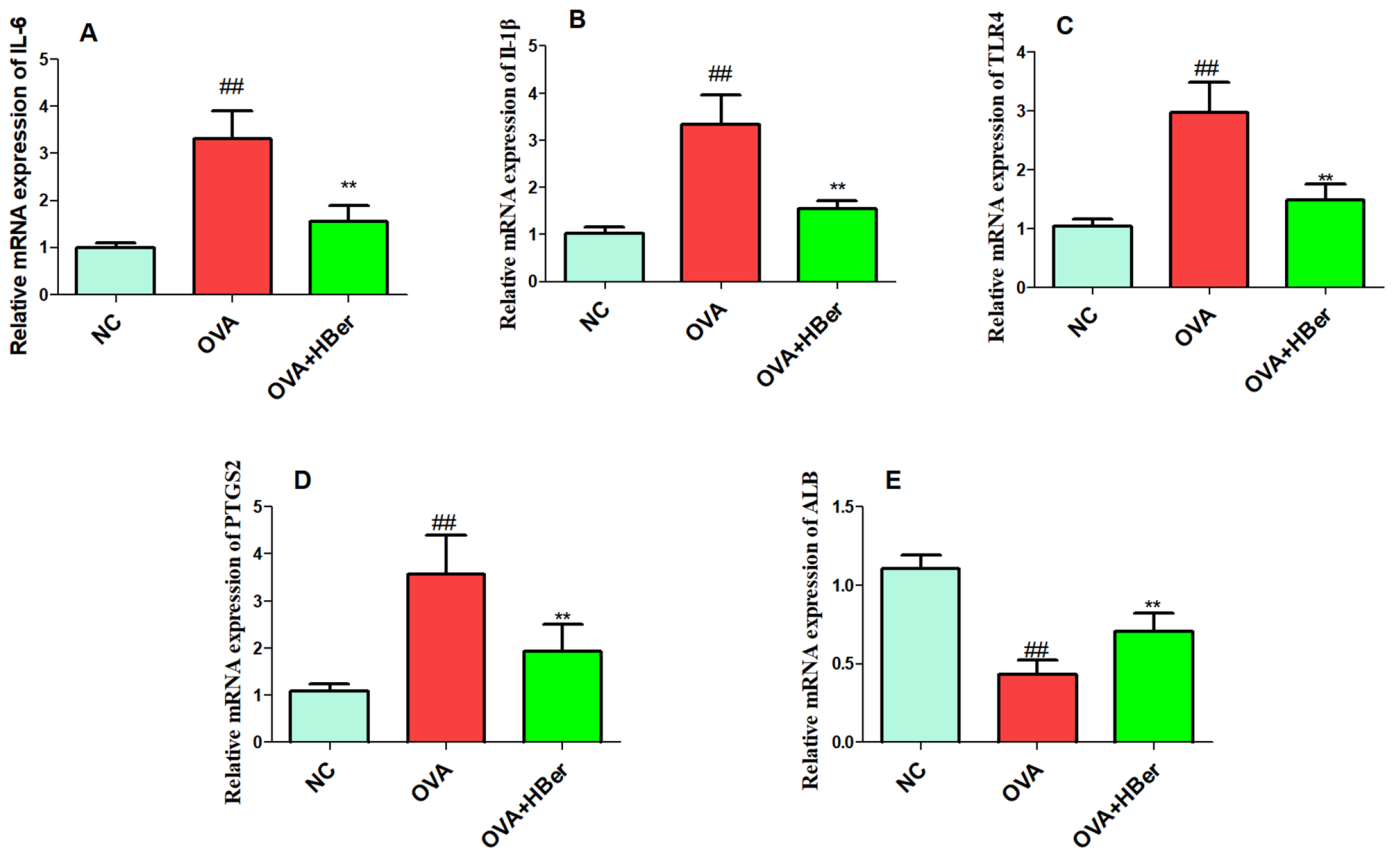
Verification of IL-6 (**A**), IL-1β (**B**), TLR4 (**C**), PTGS2 (**D**), and ALB (**E**) expression in OVA-induced allergic rhinitis. ^##^p < 0.01, the OVA group compared to the NC group; **p < 0.01, the therapeutic group compared to the OVA group. Allergic rhinitis was induced through an intraperitoneal injection of OVA (25 µg/guinea pig) three times a week for two weeks. The guinea pigs from the OVA group were treated with different doses of berberine (HBer: 100 mg/kg body weight).

The validation of molecular docking results was conducted through qRT-PCR analysis. Figure 11 illustrates that treatment with berberine (100 mg/kg) led to a significant decrease in the expressions of IL-6, IL-1β, TLR4, and PTGS2 in the OVA group, while the expression of ALB showed a notable increase in the OVA group (p < 0.01).

## Discussion

Allergic rhinitis is a chronic respiratory tract disease mediated by immunoglobulin E, characterized by symptoms such as nasal congestion and frequent sneezing^24^. This condition not only significantly impacts the patient's daily life and productivity but also worsens other symptoms like asthma^25^. Despite extensive research on treatments for rhinitis, there remains a significant lack of consistently effective treatments for a large number of patients^26,27^. Chinese traditional medicine has a long history of effectively treating allergic rhinitis symptoms^28,29^. Berberine, an alkaloid derived from Rhizoma coptidis, is known for its diverse pharmacological benefits, including anti-allergic, anti-inflammatory, and anti-tumor properties^30^. Berberine has also been shown to inhibit allergic symptoms in the ovalbumin-induced allergic rhinitis model^31^. Nevertheless, the exact mechanism through which berberine acts against allergic rhinitis remains unclear. Utilizing network pharmacological analysis is an innovative method for systematically exploring the therapeutic mechanisms of active components in diseases. Hence, this approach was employed to investigate potential interactions between target genes and active ingredients, thereby aiding in drug development^16^. The current study utilized a network pharmacology approach to uncover the potential mechanism responsible for the therapeutic benefits of berberine in treating allergic rhinitis.

Initially, 14 hub potential therapeutic targets were identified through bioinformatics analysis of the GSE52804 dataset and berberine targets. Subsequent enrichment analyses were carried out utilizing these therapeutic targets. GO-BP, KEGG, and GSEA results indicated that these targets were mainly enriched in inflammation- and immune-related pathways, the important etiological pathways of allergic rhinitis, such as inflammatory response, NF-kappa B signaling pathway, TNF signaling pathway, adaptive immune system, and cytokine signaling in immune system, etc. NF-kappa B signaling pathway is implicated in the pathologic processes of rhinitis^32^. The development of chronic inflammation in rhinitis is triggered by the intricate interplay of various cytokines, with the activation of the NF-kappa B signaling pathway driving this pathological process^33^. Activation of the NF-kappa B signaling pathway could promote IL-6 and IL-8 expression in chronic nasal sinusitis^34^. Moreover, overactive NF-kappa B signaling pathway was found to contribute to the upregulation of ICAM-1 expression, which is closely linked to the advancement of allergic rhinitis^35^. The levels of NF-kappa B expression from chronic rhinosinusitis patients were higher than that of healthy people^36^. TNF plays a crucial role as a cytokine involved in a variety of biological functions, including regulating cell death, proliferation, survival, immune responses, and inflammation^37^. TNF has the ability to induce the expression of NF-kappa B and modulate immune responses, which are crucial in the development of airway inflammation^38^. Abnormal TNF signaling is involved in the pathogenesis of various diseases, including allergic rhinitis^39,40^. Additionally, the TNF signaling pathway and the cytokine-cytokine receptor interaction were identified as key KEGG pathways in a mouse model of allergic rhinitis^41^. Berberine has been shown to deactivate the NF-kappa B signaling pathway and alleviate airway inflammation in a rat model of asthma^12^. Our analysis suggests that the TNF signaling pathway and NF-kappa B signaling pathway may be the underlying therapeutic mechanisms of berberine in treating allergic rhinitis.

Ultimately, 5 key genes (Alb, Il6, Il1b, Tlr4, and Ptgs2) were pinpointed using the R package "UpSet" and validated through molecular docking to further elucidate the intricate molecular interactions related to therapeutic targets. Among them, Il6 and Il1b are known as proinflammatory cytokines. Previous studies have indicated the involvement of cytokines in the pathogenesis of allergic rhinitis^42,43^. Il6 plays a crucial role in the development of allergic rhinitis, and its levels escalate as the disease advances^44^. Serum Il1b was identified as a potential biomarker for severe persistent allergic rhinitis^45^. Overexpression of Tlr4 is observed in individuals suffering from persistent allergic rhinitis, contributing to the advancement of allergic inflammation in this condition^46^. Additionally, in a mouse model of allergic rhinitis, the Tlr4 antagonist was found to reduce lung injury by suppressing inflammatory monocytes^47^. Ptgs2 (alias is Cox2) has been reported to play an important role in the pathophysiology of airway inflammatory response^48^. The enrichment analysis findings in this study revealed the involvement of five hub genes in the TNF and NF-kappa B signaling pathways. Moreover, molecular analysis and animal experiment were conducted to validate the network pharmacology results, demonstrating that berberine displayed strong binding affinity to Alb, Il6, Il1b, Tlr4, and Ptgs2.

While this study employs molecular docking to predict the interactions between berberine and the protein of hub genes identified in allergic rhinitis, it is imperative to acknowledge the inherent limitations and challenges of this approach: 1. Molecular docking analysis relies on computational models to predict molecular interactions, which may introduce errors and uncertainties due to the accuracy of the model and parameter settings. 2. Molecular docking analysis is typically based on static structures, while molecules in biological systems are dynamic and constantly changing, potentially leading to discrepancies between the predicted interactions and the actual biological interactions. 3. Molecular docking analysis often requires a large amount of computational resources and time, which may limit the scope and scale of the analysis.

## Conclusion

In general, the current research effectively emphasized potential pharmacological mechanisms and therapeutic targets of berberine in treating allergic rhinitis through network pharmacology and molecular docking analyses. Additionally, the therapeutic impact of berberine on allergic rhinitis may occur by influencing inflammation-related pathways such as TNF and NF-kappa B signaling pathways. This study also suggested that the integration of network pharmacology with molecular docking represents an innovative approach for identifying potential targets and mechanisms of active compounds.

### Supplementary Information

Supplementary Information 1.

Supplementary Table 2.

## Data Availability

All data can be obtained from the website we provide, and are available from the corresponding author upon request.
