# Supplementary material for: Integration of bioinformatics analysis, molecular docking and animal experiments to study the therapeutic mechanisms of berberine against allergic rhinitis
Source: Sci Rep. 2024 May 25;14:11999. doi: 10.1038/s41598-024-60871-4 (PMC11127925; doi:10.1038/s41598-024-60871-4)
Supplement: Supplementary file 1 — Supplementary Information 1. [file 41598_2024_60871_MOESM1_ESM.docx]

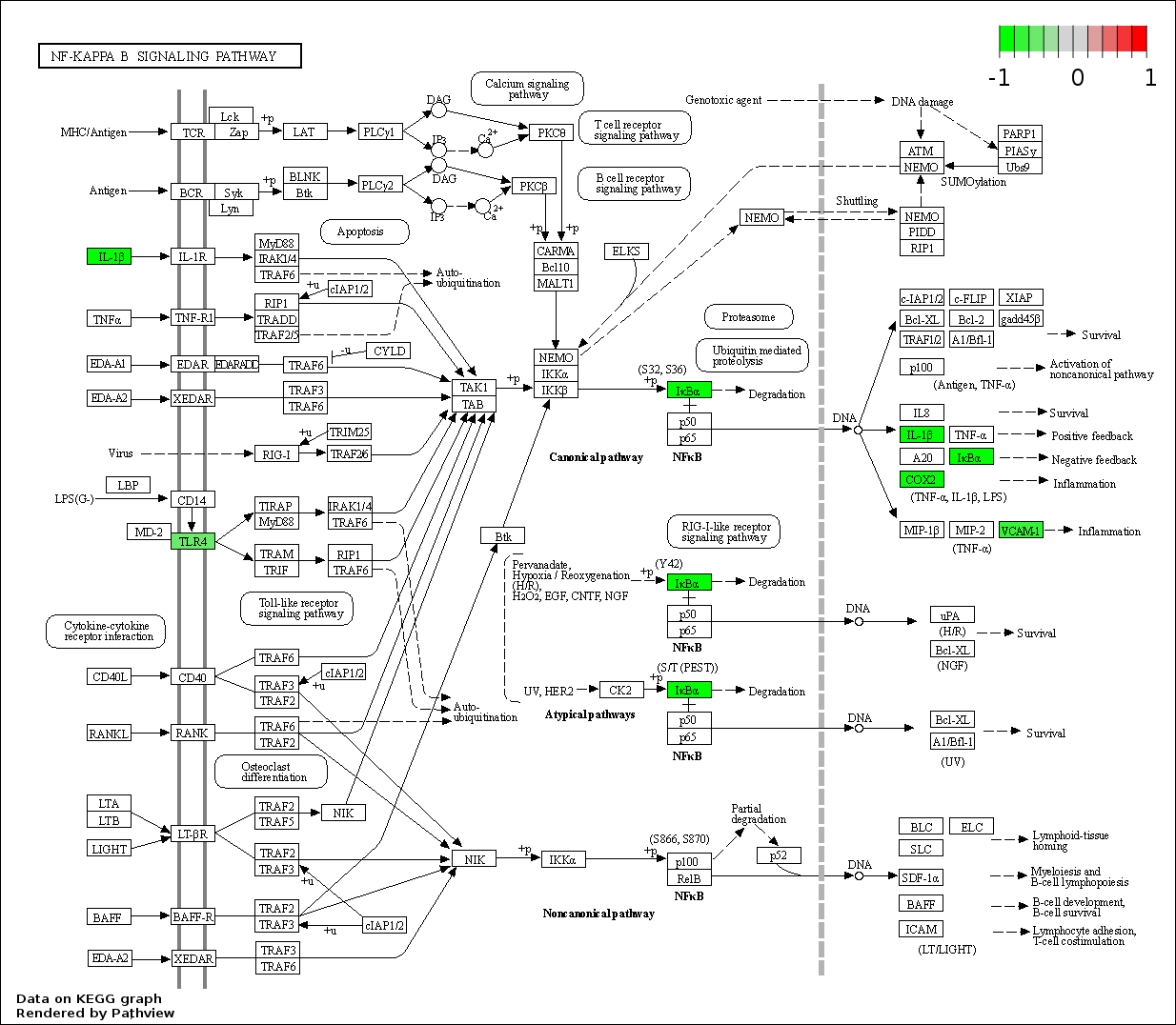


Figure S1 The potential mechanism of berberine against allergic rhinitis involved in the NF-kappa B signaling pathway. The green rectangle shows the targets associated with the PPI network.


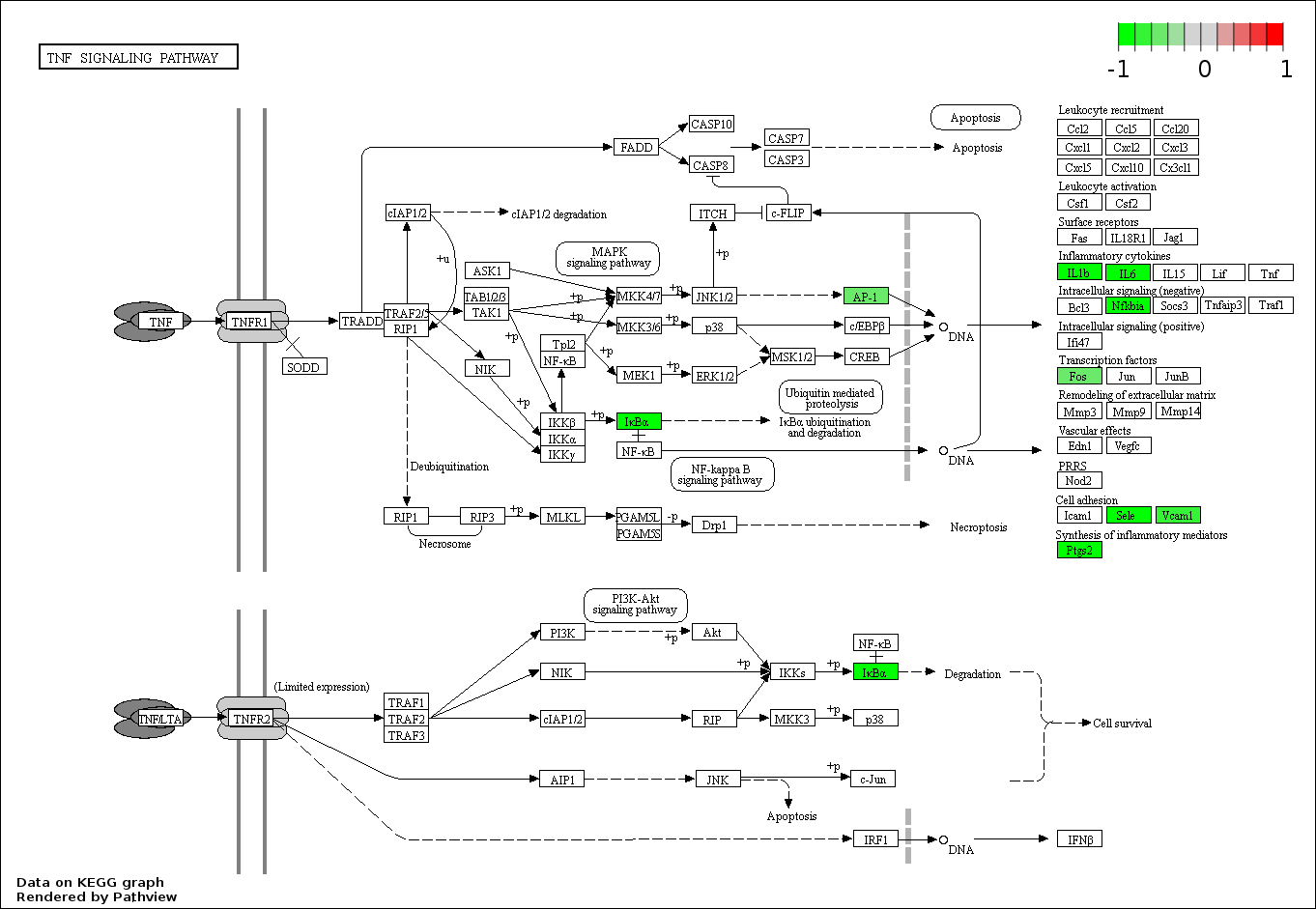


Figure S2 The potential mechanism of berberine against allergic rhinitis involved in the TNF signaling pathway. The green rectangle shows the targets associated with the PPI network.

Table S1 Sequences of primers used quantitative real-time PCR.

| Gene | Forward primer (5' to 3') | Reverse primer (5' to 3') |
| --- | --- | --- |
| TLR4 | ATCAGGACGCGCAAACATG | TGATCGCTGCTGCCTTCAC |
| IL-6 | TCCTACCCCAACTTCCAATGCTC | TTGGATGGTCTTGGTCCTTAGCC |
| IL-1β | TGAGGCTGACAGACCCCAAAAGAT | GCTCCACGGGCAAGACATAGGTAG |
| PTGS2 | CGGTGAAACTCTGGCTAGACAG | GCAAACCGTAGATGCTCAGGGA |
| ALB | GAGACCAGAGGTTGATGTGATG | AGGCAGGCAGCTTTATCAGCA |
| GAPDH | ACCACAGTCCATGCCATCAC | TCCACCACCCTGTTGCTGTA |
